# Single-cell RNA sequencing reveals the role of cell heterogeneity in the sex difference in primary hyperparathyroidism

**DOI:** 10.3389/fendo.2023.1165890

**Published:** 2023-03-07

**Authors:** Shuai Lu, Xi Chen, Maoqi Gong, Shuo Chen, Jianyu Zhang, Xigong Zhang, Chengai Wu, Aimin Cui, Xieyuan Jiang

**Affiliations:** ^1^ Department of Orthopedic Trauma, Beijing Jishuitan Hospital, Beijing, China; ^2^ Department of Adult Joint Reconstructive Surgery, Beijing Jishuitan Hospital, Fourth Clinical College of Peking University, Jishuitan Orthopaedic College of Tsinghua University, Beijing, China; ^3^ Beijing Institute of Trauma and Orthopedics, Beijing, China; ^4^ Department of General Surgery, Beijing Jishuitan Hospital, Beijing, China

**Keywords:** primary hyperparathyroidism, ScRNA-seq, sex difference, cellular interactions, gene expression

## Abstract

**Objective:**

To explore the difference in parathyroid tissue-derived cells between male and female PHPT patients.

**Methods:**

Resected parathyroid tissues were collected from PHPT patients of both sexes. Single cells were isolated and sequenced for RNA expression profiles. The cell sequencing data were annotated by cell type, followed by population analysis, functional analysis, pathway analysis, cell communication analysis, differential gene expression analysis, and pseudotime trajectory analysis. The subcluster analyses were also performed in the parathyroid cells.

**Results:**

No substantial difference in the cell population, function, or communication is found between the two sexes. The interferon-a response, oxidative phosphorylation, and reactive oxygen species pathways are up-regulated in females than in male patients, mainly contributed by fibroblast cells, endothelial cells, parathyroid cells, and myeloid cells, which also have significantly more up-regulated pathways and cellular interactions than the other three cell types. The subcluster analysis of parathyroid cells identified five subpopulations: SPARCL1-OC and ISG15-OC are predominant in females, while more S100A13-PCC and PTHLH-OC are found in males. The cellular functions are also elevated in females compared with males. Cells from female patients show a higher expression level of parathyroid hormone (PTH) but a lower expression level of parathyroid hormone-like hormone (PTHLH). The cell pseudotime trajectory and pathway analyses show that the oxyphil cells may be more mature and functionally active than the chief cells in both sexes.

**Conclusion:**

These findings suggest that the sex difference in PHPT may be caused by the differentially expressed genes and activated pathways in different cell types in the parathyroid tissue. The heterogeneity of parathyroid cell subpopulations, especially in oxyphil cells, may be associated with the sex differences in PHPT pathogenesis.

## Introduction

Primary hyperparathyroidism (PHPT) is a common endocrine disorder induced by the overproduction of parathyroid hormone (PTH) (1). Specifically, the hyperplastic parathyroid glands secrete PTH regardless of the blood calcium level, causing excessive calcium to be released from bones and kidneys into the blood, leading to hypercalcemia and a series of skeletal, renal, and neurological complications (1). Typical clinical presentations of PHPT include osteoporosis, nephrolithiasis, gastrointestinal disorders, and neurological and cognitive impairment (2). The incidence rate of PHPT ranges between 0.4 to 82 cases per 100,000 people across different races and regions worldwide (2–4). In countries and regions where blood calcium is routinely measured, such as the US and western Europe, PHPT is often diagnosed early and well managed. Therefore, most PHPT patients appear to be asymptomatic (1). In contrast, the majority of PHPT patients in Asia, South Africa, and the Middle East are symptomatic (4). Despite the regional difference, the incidence of PHPT varies significantly by sex: the female-to-male ratio in PHPT patients is approximately 3:1 (5). This sexual dimorphism appears to be wider in patients above 50 years old (6), i.e., the highest prevalence of PHPT is reported in postmenopausal women (7). Interestingly, male patients tend to be significantly younger and more frequently symptomatic than female patients, although no sex difference can be found in the levels of PTH, blood calcium, and urinary calcium. Nephrolithiasis and osteoporosis are the dominant PHPT symptoms in men and women, respectively (8). Unfortunately, little is known about the sex differences in PHPT incidence and clinical presentation. While estrogens might be potentially associated with PTH secretion, no direct evidence has been discovered (9). The role of sex in PHPT pathogenesis and outcome remains unclear, and therefore, personalized and sex-specific treatment of PHPT is still impossible to be implemented.

Single-cell RNA sequencing (scRNA-seq) is an emerging technology that enables the profiling of the transcriptomes of a heterogeneous cell population (10). Compared with the traditional bulk RNA-seq techniques, scRNA-seq allows the discovery of rare cells in a complex cell population, as well as the regulatory relationships between genes (11). Due to these unique merits, scRNA-seq has been widely applied in diverse fields, such as cancer, neurodevelopment and neurodegeneration, and hematological diseases (12–14). The parathyroid tissue has been found to be composed of three types of cells, including chief cells, oxyphil cells, and lymphocytes, with different relative abundance, genetic, and functional behaviors. Such heterogeneity may lead to alternate etiologies of PHPT, which may further translate into various clinical presentations and sex differences (15). Therefore, it might be worth investigating whether the parathyroid cells exhibit a heterogeneous transcriptome, and how it is associated with PHPT incidence, symptoms, and sex differences.

In this study, we aim to reveal the sex differences of PHPT through the scRNA-seq of dissected parathyroid tissues from PHPT patients. By annotating seven distinct cell categories, we analyzed their proportion, functions, pathways, communication, subclusters, and differentially expressed genes. We discover that the proportions of mast cells and B cells differ significantly in male and female PHPT patients. We also find in the sub-cell type analysis that PTHLH-OC and SPARCL-OC are the prominent oxyphil cells in males and females, respectively. Sex differences are also observed in the cell function, pathway, and communication analyses. Our research discovers new evidence for elucidating the sex difference of PHPT from the perspective of parathyroid cell heterogeneity, and is thus expected to provide insights into the sex-specific PHPT treatment.

## Materials and methods

### Patients and specimen

A total of 4 patients (2 males and 2 females) who underwent parathyroid resection surgery at Beijing Jishuitan Hospital were recruited in our study between January 2020 and January 2022. Signed informed consent forms were obtained from all subjects before the study. The resected parathyroid tissue samples were collected from all patients during the surgery and stored at -80 ˚C. This study was reviewed and approved by the Institutional Review Board of Beijing Jishuitan Hospital (Reg. No. 201905–01).

### ScRNA-seq of the parathyroid tissue

The single-cell suspensions were prepared from the resected parathyroid tissue as previously described. The scRNA-seq libraries were constructed using the Chromium Single Cell 3’ Reagent Kit v2 under the manufacturer’s instructions. Firstly, cells in the suspension were loaded onto a Chromium Single Cell Controller (10X Genomics, USA) to prepare the Gel Bead-in-Emulsions (GEM), in which the poly-adenylated mRNA in each cell was reverse-transcripted into cDNA encoded with a 16-bp unique sequence that varied between different cells. Next, a recovery agent was added to disrupt the emulsion. The cDNA was cleaned up with DynaBeads Myone Silane Beads (ThermoFisher Scientific, US), amplified by PCR, fragmented, end-paired, A-tailed, and ligated with an index adapter following the manufacturer’s instructions. The cDNA libraries were sequenced with a HiSeq X platform (Illumina, USA) into 150-bp paired-end read data.

### ScRNA-seq data analysis

The scRNA-seq data were analyzed following a previously described workflow (16). Data loading and quality control were performed with the R package Seurat (v 4.0.1) (17). The data of cells that met any of the following conditions were discarded before the analysis (1): The total unique molecular identifier (UMI) count was below 1,200 (2); The total number of genes was below 300 (3); The mitochondrial gene proportion was more than 20%. Based on these criteria, 16,474 cells were selected for further analysis, with 5,726 and 10,748 cells from male and female patients, respectively. Data integration was performed using the R package Harmony (Version 0.1) with default parameters (18). A total of 2,000 high variable genes were screened. The dimension reduction was performed using the top 30 principal components (PCs). For optimal cell clustering, the resolution for visualization was set to 0.2.

### Pseudotime trajectory analysis

For pseudotime trajectory analysis, R packages Monocle3 (Version 1.2.9) and Monocle (Version 2.18.0) were utilized separately with their default parameters (19). The root point was determined with the graph learning approach.

### Cell communication analysis

The scRNA-seq data were processed by the R package CellChat (Version 1.4.0) to investigate cell communication and interaction. From the CellChatDB database, we selected a total of 1,939 verified molecular interactions for cell communication analysis (20).

### Functional enrichment analysis

We used the R package ClusterProfiler (Version 4.0) to perform the functional enrichment analysis through the Gene Ontology (GO) and Kyoto Encyclopedia of Genes and Genomes (KEGG) databases (21). We scored the gene signatures using the following R packages: UCell (Version 1.3), singscore (Version 1.2.2), AUCell (Version 1.12.0), GSVA (Version 1.38.2), and irGSEA (Version 1.1.3) (22–25).

### Statistical analysis

We performed all statistical analyses with the R software (Version 4.0.5). The scatter plots were generated by the R package ggstatsplot (version 0.7.1). P-values below 0.05 were considered statistically significant in this study.

## Results

### Cellular contribution analysis

**Figure 1:**
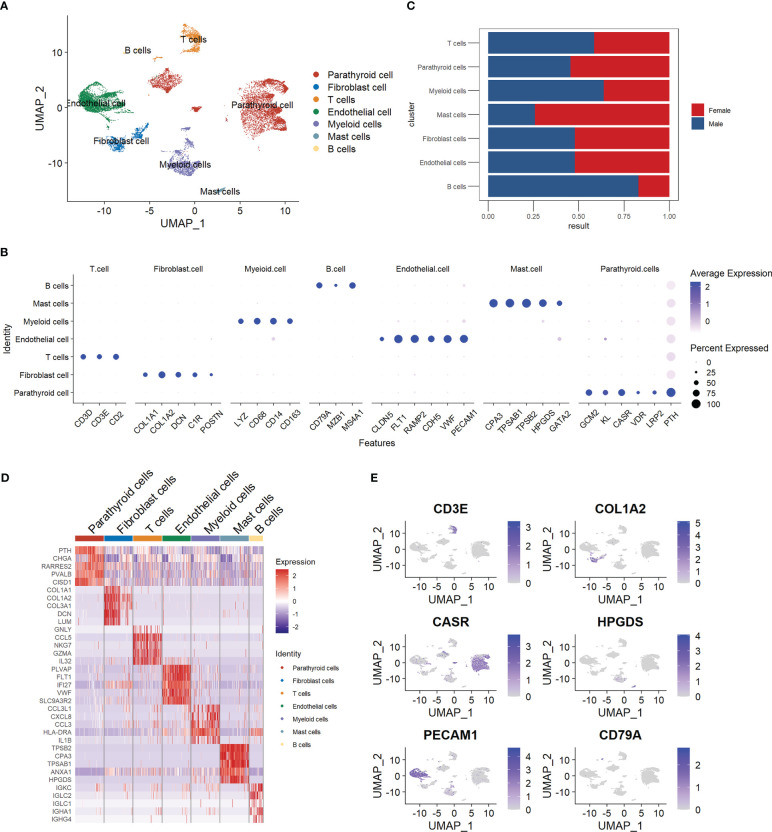
Summary of cell composition in PHPT patient samples. **(A)** Umap visualization of the seven manually annotated cell populations. **(B)** Dot plot of representative cell markers of each cell type. **(C)** Cell proportion comparison between male and female patient samples. **(D)** Heatmap of top five markers of each cell type. **(E)** Feature plot of representative cell markers of each annotated cell type.

We manually annotated the parathyroid cells into seven major cell types, including parathyroid cells, fibroblast cells, T cells, endothelial cells, myeloid cells, mast cells, and B cells. The clustering results are visualized with Umap, as shown in **Figure 1A**. **Figure 1B** depicts the expression profiles of the characteristic markers of each cell type, agreeing well with the clearly separated cell populations in **Figure 1A**. **Figure 1C** compares the cell proportions in male and female patients. It can be observed that female patients have a higher proportion of mast cells but a lower proportion of B cells compared with male patients, while there is no significant difference in other cell types. **Figure 1D** shows the expression profiles of top genes across cell types. Interestingly, in addition to the five markers used for the annotation of parathyroid cells (GCM2, KL, CASR, VDR, LRP2, and PTH), we find that CHGA, RARRES2, PVALB, and CISD1 are also highly expressed, suggesting their potential as specific markers for parathyroid cells. **Figure 1E** displays the localizations of cells expressing typical markers on the Umap, demonstrating the high annotation accuracy in detail.

### Cell functional state and pathway analysis

**Figure 2:**
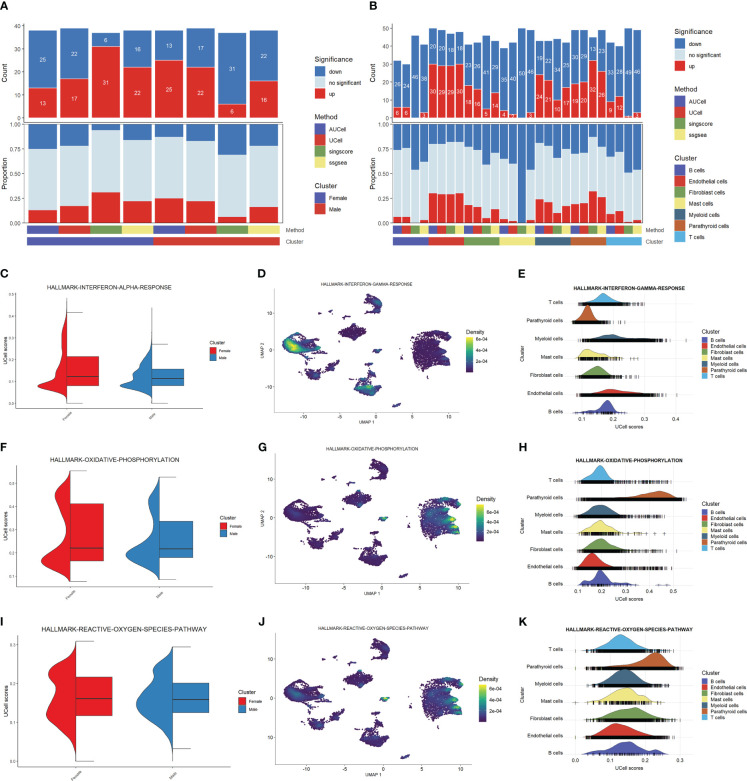
Functional analysis of PHPT patient samples. **(A)** Bar plot of the count and proportion of significant regulation pathways based on four different algorithms by sex. **(B)** Bar plot of the count and proportion of significant regulation pathways based on four different algorithms by cell type. **(C-K)** Pathway analysis of three key PHPT-related pathway, including interferon **(C–E)**, oxidative phosphorylation **(F–H)**, and reactive oxygen species **(I–K)**. Violin plots compare the UCell scores of these pathways between the two sexes **(C, F, I)**. Feature plots based on Umap show the pathway score distribution across all cell types **(D, G, J)**. Ridge maps show the pathway scores of each cell type **(E, H, K)**.

We explored the cell functions based on the pathway analysis. **Figure 2A** shows the numbers and proportions of differentially-expressed genes as evaluated by four algorithms, including AUCell, Ucell, singscore, and ssgsea. The results of these algorithms are inconsistent when comparing the male and female groups: AUCell and Ucell find more up-regulated pathways in males than in females, while singscore and ssgsea show an opposite trend. This may indicate that the cellular functions do not differ significantly between the two sexes. However, these algorithms show similar trends when the cells are grouped by cell type (**Figure 2B**). Parathyroid cells, endothelial cells, fibroblast cells, and myeloid cells have higher numbers of up-regulated pathways compared with other cell types. These results may suggest that these four types of cells are the principal functional cells in PHPT development.

We then sought to identify the pathways differentially regulated between males and females. As shown in **Figures 2C, F, I**, the interferon-a response, oxidative phosphorylation, and reactive oxygen species pathways are up-regulated in female than in male patients. Mapping these pathways to cells shows that these differences may be attributed to certain cell types (**Figures 2D, E, G, H, J, K**). Specifically, the interferon-a response pathways may be attributed to myeloid cells and endothelial cells, whereas the oxidative phosphorylation and reactive oxygen species pathways may be attributed to parathyroid cells. Fibroblast cells may also have contributed to the reactive oxygen species pathway upregulation. Taken together, these results suggest that these four types of cells may serve as the key regulatory cells in PHPT pathogenesis and play essential roles in the sex differences of PHPT.

### Cell communication analysis

**Figure 3:**
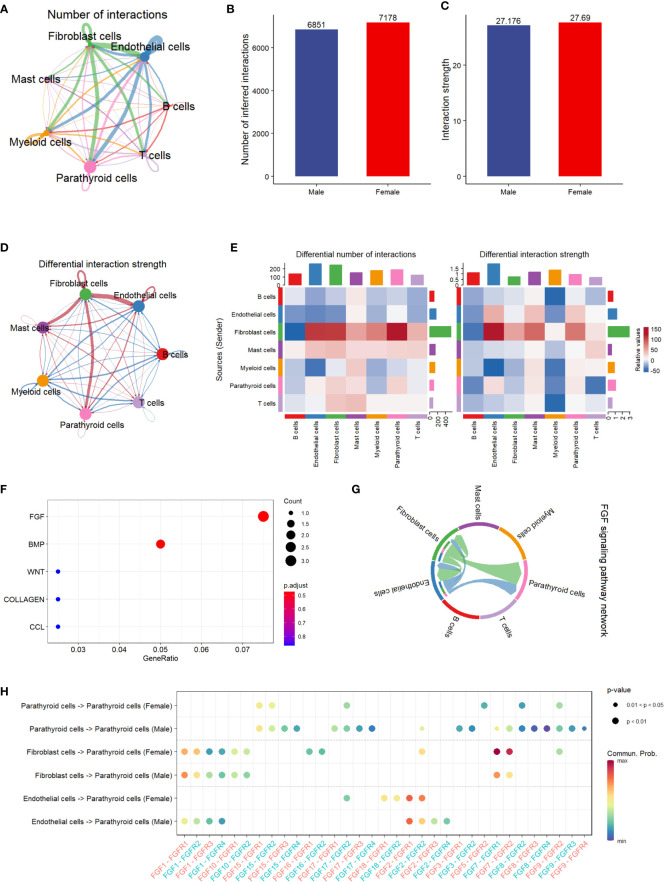
Cell communication analysis of PHPT patient samples. **(A)** String plot of the aggregated cell-cell communication network, showing the number of interactions between cell types. **(B)** Comparison of the number of interactions between male and female patients.ns=not statistically significant (unpaired t-test). **(C)** Comparison of the interaction strength between male and female patients. ,ns=not statistically significant (unpaired t-test). **(D)** Differential interaction strength comparison between male and female patients. **(E)** Heatmap of interaction numbers and strength in different cell types. **(F)** Pathway enrichment analysis of genes differentially expressed in two sexes. **(G)** FGF signaling pathway network of all cell types. **(H)** Enriched receptor-ligand pairs between parathyroid cells and other cell types in the FGF pathway.

The results of the cell communication analysis are shown in **Figure 3**, showing a similar trend to the pathway analysis. As shown in **Figure 3A**, the interactions between parathyroid cells, endothelial cells, fibroblast cells, and myeloid cells are predominant compared with those between other cell types. However, there is no significant difference between male and female patients in either the number of interactions or the interaction strength (**Figures 3B, C**). By visualizing the differential interaction strength by cell type between sexes (**Figure 3D**), i.e., subtracting the interaction strength of males from that of females, we find that the interactions related to fibroblast cells are dominant compared with all interactions involving other cell types. This is further confirmed in **Figure 3E**, as the number of interactions and interaction strength from fibroblast cells far outnumber those from all other cell types in females than in males. **Figure 3F** shows the top five up-regulated pathways related to cell communication, among which the FGF pathway is identified as the most activated pathway. As shown in **Figure 3G**, fibroblast cells and endothelial cells are the major cell types that send regulatory signals through the FGF pathway to themselves, as well as to the parathyroid cells. In **Figure 3H**, we show in detail the sender-receiver interactions between FGFs and FGFRs in different cell types and sexes. It can be observed that the FGF7-FGFR1 and FGF7-FGFR2 communications from fibroblast cells to parathyroid cells show higher probabilities in females than in males, which might be associated with the sex differences.

### Subcluster analysis of parathyroid cells

**Figure 4:**
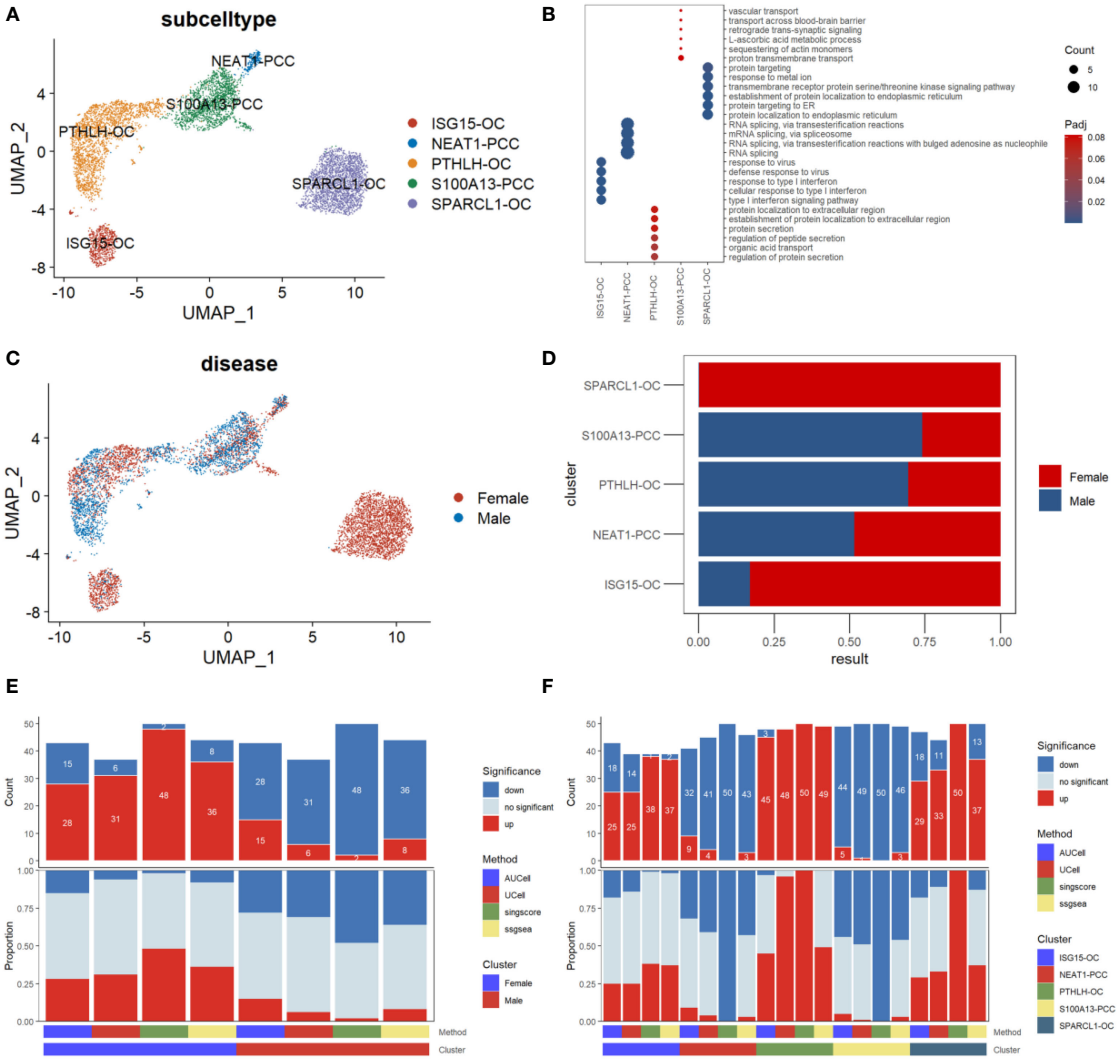
Subpopulation analysis of parathyroid cells. **(A)** Umap visualization of parathyroid subpopulation **(B)** Dot plot of representative cell markers of each cell subtype. **(C)** Umap visualization of cells derived from male and female patients. **(D)** Cell subpopulation proportion comparison between male and female patients. **(E)** Bar plot of the count and proportion of significant regulation pathways based on four different algorithms by sex. **(F)** Bar plot of the count and proportion of significant regulation pathways based on four different algorithms by cell type.

We then performed a subcluster analysis of the parathyroid cells and identified five subtypes, including two types of parathyroid chief cells (S100A13-PCC and NEAT-PCC) and three types of oxyphil cells (ISG15-OC, PTHLH-OC, and SPARCL-OC), as shown in **Figure 4A**. The expression levels of subtype-specific markers are shown in **Figure 4B**. An intriguing finding is that the subpopulations of parathyroid cells differ significantly between females and males: SPARCL1-OC and ISG15-OC are predominant in females (especially SPARCL1-OC, which is exclusively found in females), whereas more S100A13-PCC and PTHLH-OC are found in males (**Figures 4C, D**). Functional analysis reveals that the parathyroid cells in females are up-regulated compared with those in males, as predicted by all four scoring algorithms (**Figure 4E**). This may indicate that the female parathyroid cells may have a higher level of functional activation than males. In the functional analysis by parathyroid cell subtype, we find that the oxyphil cells (ISG15-OC, PTHLH-OC, and SPARCL-OC) have more significantly up-regulated pathways than the chief cells in PHPT pathogenesis (**Figure 4F**). (S100A13-PCC and NEAT-PCC), suggesting that the oxyphil cells may play more important roles than the chief cells in PHPT pathogenesis. In addition, we also calculated the Simpson index of overall cell proportion and parathyroid cells proportion separately. Compared with male, female have a lower heterogeneity of overall cells (male, 0.75; female, 0.70) but higher heterogeneity in parathyroid cells (male,0.55; female, 0.69).

### Differential gene expression analysis

**Figure 5:**
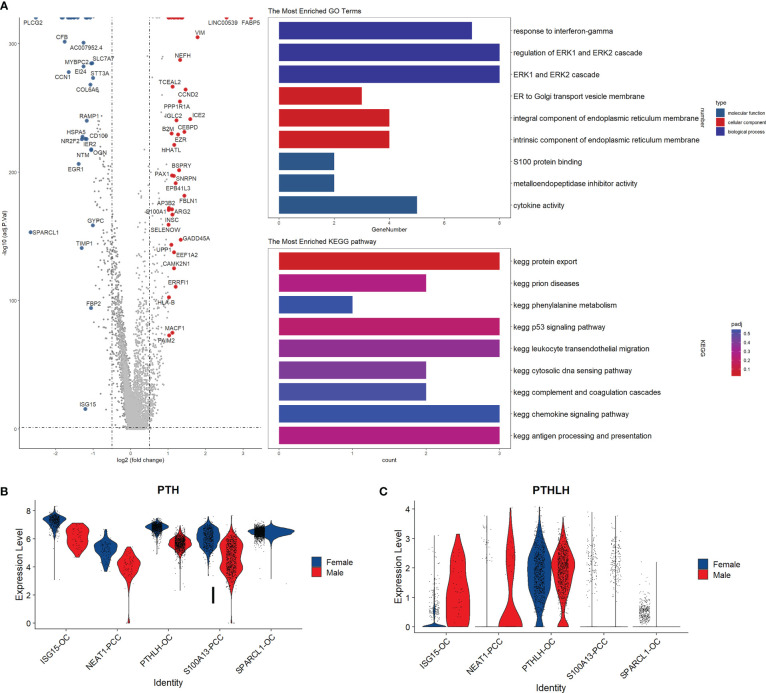
Subpopulation analysis of parathyroid cells. **(A)** Volcano plot and functional enrichment analysis of the differential expressed genes between female and male patients. Red points represent the significantly up-regulated genes in males compared with females (adjust-p<0.01, logFC>1). Blue points represented the significantly down-regulated genes in males compared with females (adjust-p<0.01, logFC<-1). **(B, C)** Expression levels of PTH and PTHLH in different cell subpopulations.

By performing the differential gene expression analysis, we identified 78 differentially expressed genes between male and female parathyroid cells, including 41 up-regulated genes and 37 down-regulated genes (**Figure 5A**). Compared with males, the top up-regulated genes in females are FAB5, VIM, and NEFH, while the top down-regulated genes in females are CFB, SLC7A7, and PLCG2. Functional analysis reveals that these up-regulated genes are associated with interferon-gamma response and estrogen transportation. It can be observed from **Figure 5B** that PTH is expressed in all cell subpopulations in both sexes, but elevated in females compared with males. In contrast, the expression level of parathyroid hormone-like hormone (PTHLH) is increased in the males compared with females, as shown in **Figure 5C**.

### Cell pseudotime and communication analysis

**Figure 6:**
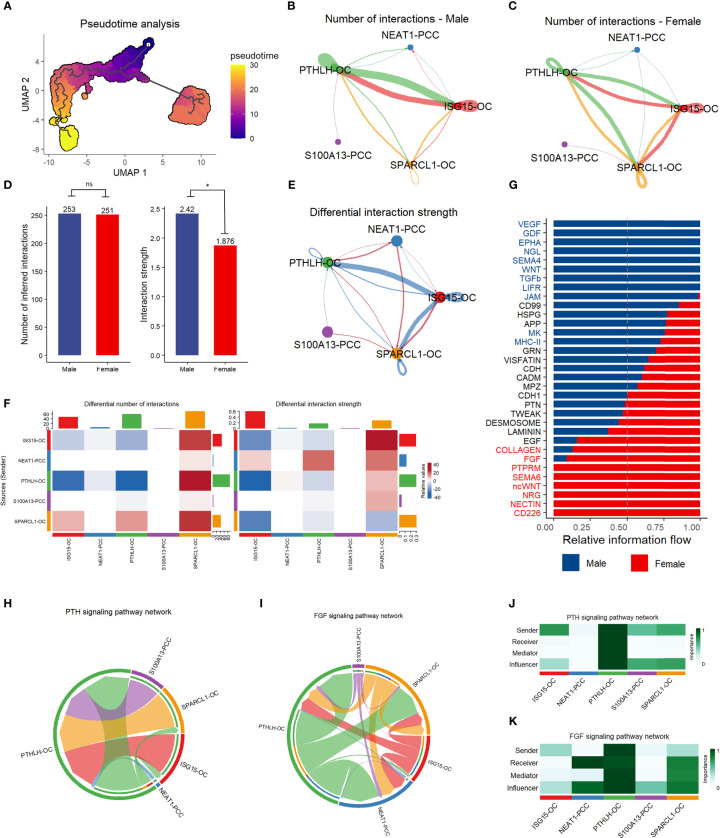
Pseudotime trajectory analysis and communication analysis of parathyroid cells. **(A)** Pseudotime trajectory analysis of parathyroid cell developmental stages. The colormap shows the pseudotime trajectory **(B, C)** String plot of the aggregated cell-cell communication network of male **(B)** and female **(C)** parathyroid cells. **(D)** Interaction number and strength comparison between male and female patients *= p < 0.05, ns, not statistically significant (unpaired t-test). **(E)** Differential interaction strength comparison between male and female patients. **(F)** Heatmap of interaction numbers and strength in different cell subpopulations. **(G)** Pathway comparison between male and female patients by parathyroid cell subpopulation. **(H, I)** String plot of PTH **(H)** and FGF **(I)** signaling pathway networks. **(J, K)** Heatmap of PTH **(J)** and FGF **(K)** signaling pathway networks.

We conducted pseudotime trajectory analysis to investigate the cell differentiation process in the parathyroid tissue. As shown in **Figure 6A**, the oxyphil cells are more mature than the chief cells, suggesting that the oxyphil cells might serve as the major functional cells in the parathyroid and may be more closely related to PHPT. Cell communication analysis reveals that oxyphil cells contribute more to cell interaction than chief cells in both male and female patients (**Figures 6B, C**). Compared with females, parathyroid cells in males have a similar number but a slightly higher strength of cell interaction (**Figure 6D**). By performing the differential analysis, it can be observed that communications between oxyphil cells are the major contributors to the apparent difference in interaction strength between sexes (**Figures 6E–G**). The analyses of PTH and FGF signaling pathway networks (**Figures 6H, I**), as well as the weight analyses by cell subtype (**Figures 6J, K**), suggest that PTHLH-OC plays a central role in the communications through both pathways and may be the major contributor for PTH and FGF production.

## Discussion

In this study, we revealed the sex differences in PHPT patients at the transcriptome level using scRNA-seq. Specifically, we investigate the cell population composition, cell functional state, and communication in resected parathyroid tissues from male and female patients. We further identify seven subclusters in parathyroid tissue and analyze their differentially expressed genes, pathways, and communications with regard to sexes.

Overall, no substantial difference in the cell population or cell communication is found between the two sexes. However, by analyzing the contributions by cell type, we show that fibroblast cells, endothelial cells, parathyroid cells, and myeloid cells have significantly more up-regulated pathways and cellular interactions, especially the fibroblast cells and endothelial cells. Furthermore, in the pathway analysis, we find that the interferon-a response, oxidative phosphorylation, and reactive oxygen species pathways are more up-regulated in females than in males. The up-regulated pathways are also mainly attributed to the aforementioned four cell types. Whereas previous studies mainly focused on the parathyroid cell and lymphocyte populations (15), our findings suggest that the fibroblast cells and endothelial cells are functionally active in the parathyroid and may play essential regulatory roles. These two cell populations may also be related to the sex difference between PHPT patients, which requires further investigation.

The cell population composition, functional state, and communication in parathyroid tissue play an important role in the pathogenesis of primary hyperparathyroidism (PHPT). The clinical significance of understanding these cellular characteristics lies in the potential to identify new therapeutic targets and improve patient outcomes. Moreover, understanding the differences in cellular characteristics between male and female patients with PHPT can provide insights into the mechanisms underlying the sex differences in disease incidence, progression, and response to treatment. This knowledge may enable the development of sex-specific diagnostic and therapeutic strategies. In summary, the study of cell population composition, functional state, and communication is crucial for advancing our understanding of PHPT pathogenesis and improving patient care.

An interesting finding is that PTH is widely expressed in all five subtypes of parathyroid cells, with higher expression levels in females than in males, whereas the PTHLH levels in males are higher than in females. PTH is mainly secreted from the parathyroid tissue, while PTHLH can be produced by almost all types of tissue in the body. PTH and PTHLH share the same PTH/PTHrP receptor but have different roles: PTH primarily regulates the blood calcium level, but PTHLH is involved in many other processes such as chondrocyte growth, mammary gland branching morphogenesis, and cancer (26). It has been reported that PTHLH is predominantly expressed in the oxyphil cells in the parathyroid tissue (27), but its potential implications in the sex difference of PHPT remain unclear. It would be valuable to compare the PTH and PTHLH expression levels in both sexes and further investigate their associations with PHPT development and clinical manifestations.

The subcluster analysis of the parathyroid cells also shows a substantial difference between males and females in the oxyphil cells. The SPARCL1 oxyphil cells are exclusively found in females, while the PTHLH oxyphil cells are the major oxyphil cells in males. Our findings suggest that the oxyphil cells may play more important roles than the chief cells in PHPT: the cell pseudotime trajectory and pathway analyses show that the oxyphil cells may be more mature and functionally active than the chief cells; cell communication analysis reveals that the oxyphil cells are mainly responsible for cellular interactions; the PTH signaling pathway network also suggests that the oxyphil cells contribute more than the chief cells. Our result agrees with previous studies that more PTH is mainly produced by the oxyphil cells, instead of the chief cells (15, 28). Also, it has been reported that the number of oxyphil cells increases dramatically with age and in patients with chronic kidney disease, which often leads to secondary hyperparathyroidism (29). Our findings confirm at the single-cell level that the oxyphil cells are more essential influencers than the chief cells in cellular function and communication. Also, the heterogeneity of the oxyphil cell subpopulation may lead to the dramatic difference in PHPT incidence across sexes, which needs to be confirmed in future studies.

The study found that there was no significant difference in the overall cell population, function, or communication between the two sexes. However, several pathways, including the interferon-a response, oxidative phosphorylation, and reactive oxygen species pathways, were up-regulated in females compared to males, mainly contributed by specific cell types. The subcluster analysis of parathyroid cells revealed different subpopulations in males and females. The study also found differences in the cellular functions and expression levels of certain hormones. The study suggests that the sex difference in PHPT may be caused by the differentially expressed genes and activated pathways in different cell types in the parathyroid tissue, particularly in oxyphil cells.

## Data availability statement

The datasets presented in this study can be found in online repositories. The names of the repository/repositories and accession number(s) can be found in the article/supplementary material.

## Ethics statement

The studies involving human participants were reviewed and approved by the Institutional Review Board of Beijing Jishuitan Hospital (Reg. No. 201905–01). The patients/participants provided their written informed consent to participate in this study.

## Author contributions

SL, XC and XJ conceived and coordinated the study. SL, XC and XJ designed this study. SL, XC, MG, SC, JZ, XZ, CW, and AC performed and analyzed the experiments, wrote the paper. SL, XC, XZ, CW, and AC carried out the data collection, data analysis. XJ revised the paper. All authors contributed to the article and approved the submitted version.
